# Understanding How Athletes Manage Uncertainty in Sport

**DOI:** 10.3390/bs16040616

**Published:** 2026-04-21

**Authors:** Ran Assa, Abira Reizer

**Affiliations:** Department of Psychology, Ariel University, Ariel 40700, Israel

**Keywords:** uncertainty in sport, perceptions, coping skills

## Abstract

Uncertainty is a central feature of sport and has been extensively examined in sport science, primarily from performance-oriented perspectives such as anticipation, decision-making, and motor control. However, less attention has been given to how athletes subjectively perceive and experience uncertainty and how these interpretations shape their responses. The present study addresses this gap by exploring athletes’ lived experiences of uncertainty in sport. Using a qualitative design, semi-structured interviews were conducted with former youth athletes from various sports. Data were analyzed using thematic analysis, generating 15 themes organized across key dimensions of uncertainty, including unpredictability, lack of information, internal versus external sources, and the appraisal of uncertainty as a threat or a challenge. Findings indicate that uncertainty is experienced as a multifaceted and subjective phenomenon shaped by perceived control, prior experience, and situational context. Athletes differed in how they interpreted uncertainty, with some perceiving it as threatening and others as an opportunity for growth, which in turn influenced emotional responses and coping strategies. Key coping mechanisms included communication, information seeking, social support, and focusing on controllable aspects of performance. These findings extend existing sport science literature by integrating experiential and interpretative dimensions of uncertainty with established performance-based approaches. Furthermore, the results suggest conceptual links with the construct of intolerance of uncertainty (IU), highlighting the potential value of examining individual differences in how athletes appraise and manage uncertainty. The study provides an exploratory foundation for future research integrating IU within sport contexts and underscores the importance of addressing both subjective and performance-related aspects of uncertainty in sport psychology.

## 1. Introduction

Uncertainty is a central feature of human performance and decision-making, particularly in dynamic and time-pressured environments such as sport. Athletes are frequently required to act under conditions of incomplete information, ambiguity, and constantly changing environmental constraints, which place substantial demands on their cognitive, emotional, and behavioural regulation.

Accordingly, uncertainty has been widely examined within sport science, particularly in relation to perceptual–cognitive processes such as anticipation, decision-making, and sensorimotor control. For example, research has shown how athletes integrate kinematic information and contextual priors to make predictions under uncertainty (Gredin et al., 2020) and how sensorimotor systems adapt to uncertain task constraints (Beck et al., 2025). In addition, studies on decision-making have demonstrated that the use and value of information under uncertainty are influenced by factors such as expertise and prior knowledge (Magnaguagno et al., 2022).

Uncertainty has also been examined in relation to emotional processes, particularly anxiety. For instance, recent work suggests that uncertainty plays a key role in how individuals update predictions about environmental states and regulate emotional responses (Harris et al., 2025). Together, these lines of research demonstrate that uncertainty is a well-established construct within sport science.

However, despite this growing body of literature, most research has approached uncertainty from an objective or performance-oriented perspective, focusing on how athletes process information, anticipate events, and execute actions under uncertain conditions. In contrast, considerably less attention has been paid to how athletes subjectively perceive and experience uncertainty and how these perceptions shape their emotional responses and coping strategies in real-world sporting contexts.

Conceptualizing uncertainty as a subjective experience is important, as individuals differ in how they interpret and respond to uncertain situations. In the context of the present study, uncertainty is therefore conceptualized as a subjective and phenomenological experience, reflecting an athlete’s perceived inability to predict, interpret, or control aspects of a situation. This approach aligns with qualitative perspectives that emphasize lived experience and meaning-making (Smith & Sparkes, 2016), as well as broader conceptualizations of uncertainty as a perception of the unknown (Carleton, 2012; Hillen et al., 2017). Importantly, the way athletes perceive uncertainty is likely to influence their cognitive and emotional responses, and in turn shape the coping strategies they employ. Examining both perceptions of uncertainty and coping responses may therefore provide a more comprehensive understanding of how athletes function under uncertain conditions. Given the limited understanding of athletes’ lived experiences of uncertainty, the present study adopts an exploratory qualitative approach. Specifically, this study aims to provide an initial conceptual and empirical foundation for examining uncertainty in sport from the athletes’ perspective. By doing so, it seeks to extend existing sport science literature by integrating experiential dimensions of uncertainty with established performance-oriented perspectives. Furthermore, this exploratory work is intended to inform future research that seeks to investigate uncertainty using established constructs such as intolerance of uncertainty (IU). IU has been conceptualized as a dispositional tendency to react negatively to uncertain situations (Carleton, 2012) and has been widely studied in clinical and cognitive domains. However, its applicability to sport contexts remains underexplored. The present study therefore represents a first step toward understanding how uncertainty is experienced in sport and how constructs such as IU may be meaningfully adapted and examined in this domain.

### 1.1. Defining Uncertainty

According to information theorists, uncertainty is defined as “an individual’s perceived inability to predict something accurately” (cited in Milliken, 1987, p. 136). Uncertainty is experienced when an individual perceives themselves to be lacking sufficient information or because of an inability to differentiate between relevant and irrelevant data when trying to predict events accurately (Gifford et al., 1979). Cognitive theorists, such as Kagan (1972), would suggest that uncertainty results from an inconsistency between cognitions, experience, and behaviour. For example, athletes may perceive a situation (e.g., defeat in a game) as uncertain as they cannot predict its future consequences, especially if they are feeling doubts that could lead to a possibly unpleasant experience, such as failure.

Following Kagan’s line of thought, the source of uncertainty is a key point and crucial to the way an individual perceives it; uncertainty can often be interpreted as subjective, internal, and concerning the individual’s perception. However, uncertainty could also be interpreted as external and in relation to environmental factors (Krüger & Hermsdörfer, 2019). In organizational psychology, when attached to the term “uncertainty”, “environmental” implies that the source of uncertainty is the organization’s external environment; once again, the unpredicted “something” is specified as being rooted in the organizational environment.

Milliken (1987) offered three types of environmental uncertainty regarding organizations, which can also be applied in a sports context. The first is “state uncertainty”, which postulates that managers or administrators do not understand how environmental components might be changing. In a sports context, this occurs when a manager or player is struggling to predict the behaviour of an upcoming opponent in a competition. The second type of environmental uncertainty is “effect uncertainty”, which concerns an individual’s ability to predict the impact of changes in one of the environmental components, for example, an athlete who cannot see the effects of different weather on their sporting style. Lastly, “response uncertainty” is associated with the ability to comprehend what response is available and suitable for a change in one’s environment; in this type of uncertainty, the individual lacks knowledge of response options and their consequences. Response uncertainty is common in sports as athletes need to weigh up the options available to them to react to their opponent’s behaviour all the time.

In a paper that aspired to compare organizational behaviour in industries to athletic behaviour in sports cultures, uncertainty was mentioned as “a fundamental characteristic of any complex interpersonal or socio-technical system designed to achieve performance goals” (Davids et al., 1991). Similar to manufacturing production, competitive sport seeks to optimize system performance in conditions of uncertainty. Researchers have argued that forms of control are essential characteristics for successful performance in both areas: the need to control the source uncertainty might be essential to improve performance in both organizations and high-level sports cultures (Cherns, 1976).

Finally, Michael (1973) viewed uncertainty as a loss of control regarding the self and the situation. The need for control is grounded in human nature and relies on learning to develop the self as “an internalization of the ‘generalized other’” (cited in Downey & Slocum, 1975, p. 571). Although it tends to be viewed as behaviouristic, this definition postulates that humans search for meaning due to the nature of goal-directed behaviour.

In the present study, uncertainty is conceptualized as a subjective and phenomenological experience, referring to an athlete’s perceived inability to predict, interpret, or control aspects of a sporting situation. This approach emphasizes how uncertainty is experienced and constructed by the individual, rather than assuming a solely objective definition (Carleton, 2012; Hillen et al., 2017; Smith & Sparkes, 2016). To further strengthen the conceptual grounding of uncertainty, it is important to recognize that decision-making literature differentiates between multiple forms of uncertainty, including ambiguity, risk, and volatility (Bland & Schaefer, 2012). These distinctions highlight that uncertainty is not a unitary construct but rather a multifaceted phenomenon that varies depending on the availability and reliability of information. This perspective aligns with the present study’s focus on how athletes subjectively interpret and experience uncertainty within sport contexts.

### 1.2. Uncertainty and Decision-Making Processes

Uncertainty is a central construct in the study of decision-making within mainstream psychology (e.g., Kahneman & Tversky, 1973). In naturalistic settings, uncertainty constitutes a significant obstacle to effective decision-making, particularly when individuals must act under time pressure and incomplete information (Gréhaigne et al., 2001; van den Heuvel et al., 2014). Decision theorists have emphasized the role of subjective judgments and probabilistic reasoning in such contexts, highlighting the importance of how individuals interpret and respond to uncertain situations (Downey & Slocum, 1975). More recent research in sport and motor control has examined how uncertainty influences decision-making at a perceptual–motor level. For example, studies suggest that athletes integrate contextual and kinematic information to guide action under uncertainty (Gredin et al., 2020), while sensorimotor systems adapt dynamically to uncertain task constraints (Beck et al., 2025). In addition, the use and value of information in decision-making under uncertainty appear to be shaped by expertise and prior knowledge (Magnaguagno et al., 2022). From a motor decision-making perspective, uncertainty may arise from different sources. Krüger and Hermsdörfer (2019) distinguish between internal (free choice) and external (forced choice) uncertainty. External uncertainty is associated with incomplete environmental information, requiring individuals to act before sufficient data are available, whereas internal uncertainty reflects ambiguity between multiple response options. In sporting contexts, athletes frequently encounter both forms, as they must select actions in real time while weighing multiple possible outcomes. Taken together, these findings suggest that uncertainty plays a central role in decision-making processes in sport. However, existing research has largely focused on observable behaviour and performance outcomes, with less attention given to how athletes subjectively experience and interpret uncertainty during decision-making.

### 1.3. Sports Environment and Demands for Athletes

Sporting environments are inherently dynamic, time-constrained, and information-rich, requiring athletes to make rapid decisions under pressure (Gréhaigne et al., 2001). These demands are further intensified by contextual factors such as competition level, audience presence, and performance expectations, all of which contribute to increased uncertainty (Macquet, 2009). Differences in decision-making between expert and novice athletes have been widely documented, with expertise associated with more efficient information processing, anticipation, and response selection (Gréhaigne et al., 2001). More recent research suggests that skilled athletes are better able to utilize contextual information to reduce uncertainty and guide action (Gredin et al., 2020), highlighting the role of experience in shaping responses to uncertain situations.

In addition to cognitive and perceptual demands, psychological characteristics such as mental toughness have been identified as critical for performance under pressure. Elite performers are often described as being able to adapt to and cope with unplanned and uncertain situations (Jones et al., 2007). These capabilities include maintaining focus, regulating emotions, and making effective decisions despite ambiguity and time constraints. Together, these findings indicate that uncertainty is not only inherent in sport but also central to performance. However, while existing research has primarily examined how athletes perform under such conditions, less is known about how they perceive and interpret these uncertain environments.

### 1.4. Uncertainty and Anxiety

Uncertainty has been prevalent in information theory and other domains in psychology (i.e., cognitive, organizational) but not particularly in theorizing about anxiety. It has been identified as a critical stimulus to anxiety related to disruption in performance in complex systems involving high information load and demonstrated to be a characteristic of performance-related information (Davids et al., 1991). A sporting environment is loaded with information as athletes need to perform in front of large, noisy and distracting crowds and must cope effectively with long journeys and problems with training facilities and accommodation. These aspects could increase this load and create uncertainty among athletes (Macquet, 2009). Added pressures, such as fans’ expectations, the financial cost of failure, and media coverage, only increase the challenge for athletes as the competitive environment is highly demanding and risky (Davids et al., 1991). Future events that could not be expected, or the uncertainty of the future, have been postulated to be anxiety-provoking by psychologists throughout the last century. According to Kagan (1972), the resolution of uncertainty is a fundamental motive in human nature, and the process of dealing with that can be extremely threatening; if the appropriate response to resolving uncertainty is not available immediately, distress or anxiety may occur. Thus, people are constantly attempting to act in a way that reduces their own uncertainty while trying to avoid negative affect (FeldmanHall & Shenhav, 2019).

However, people tend to perceive different levels of uncertainty in different ways, as not all of them should be expected to be perceived as threatening; according to McGrath (1970), there is an individual threshold that varies between humans. In competitions, uncertainty is inevitable, and whether an individual will perceive it as positive and challenging or negative and threatening depends on their perception (Martens et al., 1990). The uncertainty of the outcome and the importance of the outcome are the two constructs proposed to explain how different objective competitive situations affect a person’s perception of a threat and thus provoke anxiety. Contemporary research suggests that uncertainty plays a central role in how individuals update predictions about environmental states and regulate emotional responses (FeldmanHall & Shenhav, 2019; Harris et al., 2025). In sporting contexts, this may manifest in athletes’ anticipatory thoughts about performance outcomes, social evaluation, and potential failure. Importantly, individuals differ in how they perceive and respond to uncertainty. While some may interpret uncertain situations as threatening, others may perceive them as challenging or motivating (Martens et al., 1990). These differences in perception are likely to influence emotional responses such as anxiety, as well as subsequent coping strategies. Thus, understanding how athletes perceive uncertainty may be critical for understanding variability in anxiety responses and performance in sport.

### 1.5. Reducing Uncertainty

When uncertainty exceeds an optimal level, individuals are motivated to reduce it through various cognitive and behavioural strategies (Kagan, 1972). A primary mechanism for reducing uncertainty involves information-seeking, aimed at increasing predictability and narrowing the range of possible outcomes (FeldmanHall & Shenhav, 2019). In social contexts, Festinger’s (1954) social comparison theory suggests that individuals seek information from others to evaluate their abilities when objective standards are unavailable. In sport, this may involve gathering information about opponents, performance conditions, or expectations prior to competition. Coping with uncertainty may also involve internal strategies, such as focusing on controllable aspects of performance or relying on prior experience. Research suggests that experienced athletes are better able to anticipate events and interpret available information, thereby reducing perceived uncertainty and associated anxiety (Martens et al., 1990; Gredin et al., 2020). Beyond adaptation to task constraints, recent work has highlighted additional mechanisms such as risk optimization and redundancy exploitation, whereby performers adjust their behaviour to maintain performance stability under uncertain conditions (Beck et al., 2025). These mechanisms further illustrate how athletes actively regulate uncertainty, complementing the experiential and interpretative accounts identified in the present study. However, while these strategies have been examined in relation to performance and decision-making, less is known about how athletes themselves perceive and describe these processes in real-world sporting contexts.

## 2. Uncertainty in Sport and the Current Study

The sport science literature has demonstrated that uncertainty is relevant to anticipation, decision-making, sensorimotor control, and emotional regulation in sport. However, less is known about how athletes themselves perceive, interpret, and cope with uncertainty as part of their lived sporting experience. Accordingly, the present study adopts an exploratory qualitative approach to examine athletes’ perceptions and experiences of uncertainty in sport. This exploratory framing is particularly appropriate given the study’s broader aim of providing an initial conceptual foundation for future work on intolerance of uncertainty (IU) in sport contexts.

The purpose of this study was therefore to better understand how athletes perceive uncertainty in sport and how they cope with it. Specifically, the study had two aims: (a) to explore the ways athletes perceive uncertainty in sport and (b) to analyse the coping strategies they use to manage uncertain situations.

## 3. Methods

### 3.1. Research Design

A qualitative research methodology was selected for the current study due to its benefits regarding the purpose of the research topic. Qualitative research offers researchers an opportunity to examine things in their natural settings and allows participants to give meaning to their interpretation(s) of the phenomenon under study (Smith & Sparkes, 2016). Although the literature has indicated differences between the ways people perceive uncertainty, it is important to understand athletes’ perceptions and the mechanisms that contribute to the generation of uncertainty (e.g., van den Heuvel et al., 2014; Kramer, 2014). Qualitative research has become more prominent in revealing such differences as more diverse methods have become increasingly widely accepted in the sports psychology literature.

### 3.2. Participants

Four male and four female former youth athletes (aged 22–25) formed the sample for this study, from the UK. All the participants are currently students and agreed to participate in the study after fulfilling the minimum criteria of at least five years’ competitive experience as a national (e.g., top division or state championship) or international (e.g., Commonwealth Games) athlete. The sports represented were fencing, football, netball, and Gaelic football. The participants had an average of 7.5 years of national experience. Three participants competed at an international standard during their careers, and the remaining five participants were former national athletes; some are currently semi-professional athletes. Although the sample size is relatively small, it is consistent with qualitative research aiming to explore in-depth subjective experiences (e.g., Landrum & Davis, 2023). The focus of the present study was on gaining rich, detailed insights into how athletes perceive and cope with uncertainty. To enhance transparency and comparability across studies, participants can be broadly classified according to the participation-based “Tier” framework proposed by McKay et al. (2021). Based on this framework, the current sample may be characterized as predominantly recreational to trained athletes, providing important context for interpreting the findings.

### 3.3. Interview Guide

In order to elicit the relevant data and facilitate the interview process, a semi-structured interview guide was developed. It included a sequence of questions with a flexible structure regarding uncertainty. In general, the questions focused on the individual perceptions of uncertainty in sport, what mediators, in the respondent’s opinion, contribute to generating uncertainty in sport, and the mechanisms that helped them cope with moments of uncertainty. The questions chosen were based on the literature in the context of uncertainty that emphasizes the subjective experience of uncertainty and the several sources that generate it. Questions regarding dealing with uncertainty were open-ended as they provided the athletes the opportunity to elaborate on their own points of view. The semi-structured interview guide focused on the process(es) an athlete goes through when encountering uncertain events during their sporting career, the emotions that accompany those moments, and their cognitive process after facing uncertainty. The interview guide used in this study will be provided as Supplementary Material to enhance transparency and support replication.

### 3.4. Procedure

First, the participants were invited to take part in this research. Eight people volunteered to proceed and schedule a time for an interview after reading the information sheet attached to the email with the research topic. In line with Ariel University’s ethical research guidelines, the interview process, their rights, how the information from the interview would be used, how confidentiality would be maximized, and the aims and objectives of the study were outlined to the participants. A consent form was then obtained from all participants before each interview. All interviews were conducted face-to-face by the researcher. All interviews were audio-recorded in their entirety and transcribed verbatim. Each interview lasted approximately 50 min, and collectively, the interviews yielded over 60 typed pages.

### 3.5. Data Analysis

A six-phase thematic analysis, as used by Braun et al. (2016), was adopted in this study in order to identify the meaning of the dataset. The phases are presented here sequentially, but the actual process was recursive as the researcher moved back and forth between the phases. In the first phase, the author enhanced his familiarity with the data by reading the transcripts repeatedly. In addition to reading the transcripts, the author also developed initial ideas that emerged from the data and represented the data’s basic units. The second phase was coding, which involved systematically and thoroughly reviewing relevant aspects of the dataset. The coding phase involved the production of initial codes, whether semantic or latent content, derived from the data and general theory (Braun et al., 2016). The third phase included searching for themes as the author clustered these codes into themes representing higher-level patterns across the data. In this phase, the author started thinking about the relationships between the codes found and between different levels of themes and sub-themes, which represented common aspects of the participants’ experience. These themes were categorized and then reviewed in phase four in order to check whether they fitted with the raw dataset and addressed the purpose of the study coherently. The themes were completed with a rich analytic narrative in phase five before being written up in the final phase.

### 3.6. Rigor and Trustworthiness

After the author completed this initial analysis, a staff member was provided with his interpretation of the results and a selection of individual transcripts and asked to comment critically. The purpose of this important procedure was to judge the qualitative research’s quality and formed part of the author’s intention to enhance its trustworthiness (Smith & Sparkes, 2016). In order to enhance confirmability, according to Smith and Sparkes (2016), a researcher can use a “critical friend” (a staff member in the current study) to identify and report on a researcher’s influence (background, perceptions, procedures, etc.) on the data analyzed from transcriptions in order to produce comprehensible and trustworthy data. The author also used peers to review the accuracy of the analysis and identify parts of the data that did not support themes that emerged from the data (negative case) in order to increase credibility (Smith & Sparkes, 2016).

## 4. Results and Discussion

This study intended to identify how athletes perceive uncertainty in sport, what mechanisms or processes are related to this uncertainty, and how sportspeople cope with uncertainty. The analysis identified 15 sub-themes, which were organized into several higher-order themes. These higher-order themes include (a) inability to predict future events, (b) internal versus external sources of uncertainty, (c) perception of uncertainty as a threat or challenge, (d) uncertainty in decision-making, and (e) lack of information as a source of uncertainty.

### 4.1. Perceived Uncertainty

The first step in exploring how uncertainty is perceived among athletes is to discover how an individual athlete interprets uncertainty due to the multiple definitions of the term mentioned in the introduction. The participants interpreted uncertainty in different ways and mentioned five main themes regarding perceiving uncertainty, which will be discussed below: their inability to predict future events, the difference between internal and external uncertainty, perceiving uncertainty as a threat or a challenge, uncertainty in decision-making, and a lack of information as a source of uncertainty. It is essential to say that the participants described the first meaningful situation in which they experienced uncertainty, and for most of them, it was a negative moment for which they were unprepared.

#### 4.1.1. Inability to Predict Future Outcomes

The first theme offered by all the participants regarding the way they perceive uncertainty was their inability to predict future events. The meaning of uncertainty, in their view, is that events on and off the pitch can happen with specific levels of probability, but the fact that they cannot be sure about which possibility will occur, or even if it will, makes them perceive those situations as uncertain. The possibility of being selected for a team, their chances of defeating an opponent, and their chances of returning to full fitness after an injury are some of the sporting situations that the participants struggled to predict.

*It’s not knowing what comes after, and the visual that I’m imagining is a curtain and not knowing what’s behind the curtain… Maybe apply it in a football context, and just kind of not knowing what’s going to happen or not knowing what you’re getting yourself into.*

(Participant 6)

*Defining uncertainty … would be sort of along the lines of not knowing what the future holds.*

(Participant 4)

#### 4.1.2. Internal vs. External Uncertainty

The source of uncertainty, whether it is internal or external, affects the way athletes can cope with it. While trying to define uncertainty and sharing their experiences, the participants described internal and external factors that made them perceive the situation as uncertain. External factors are those in competitive surroundings that are perceived as elements inherent in competitive situations (e.g., referee decisions, playing in front of a crowded stadium), not as part of an athlete’s behaviour or the consequences of their decisions. In contrast, internal processes, such as perceptions of one’s abilities or feeling unprepared, are within the athletes’ ability to handle and were not given as external factors; nevertheless, these could still cause them to feel uncertain and disrupt their level of performance.

*The internal side was harder… I felt like I wasn’t good enough, like I wouldn’t make it as a pro) professional baseball player), and that was really hard to accept…*

(Participant 8)

*The uncertainty of trials and the political side of the game is horrific… There was a huge bias towards players and those based around [the capital city]… They wanted to pick them up regardless of whether or not you were a good player.*

(Participant 3)

One participant suggested that there is a link between internal and external factors that create uncertainty; he claimed that the way an athlete perceives an external source of uncertainty, such as refereeing decisions or spectators in the stadium, could be interpreted differently under different circumstances and thereby increase or decrease the athlete’s sense of uncertainty.

*External factors are dependent on internal ones… On one day, a big crowd and a scout being there, with your parents and coach watching, can be used as fuel for you to do your best and build huge confidence inside you to go out and perform and excel… On another day, that exact same external pressure can crush you, yet nothing’s changed externally—it’s just internal.*

(Participant 6)

#### 4.1.3. Threat vs. Challenge

When encountering an uncertain situation, the participants’ elaborations revealed two distinct ways of perceiving it: threatening or challenging. The difference between the perceptions of uncertainty affected the participants’ performance and the way they coped with the uncertainty. When viewing uncertainty as a threat, the participants’ attitude made them feel a lack of confidence and question whether the challenge was beyond their abilities. Since playing was important for them, uncertainty was perceived as a threat to their identity in the event that the situation resulted in failure.

*I think I saw it as a threat in the beginning, and it hindered me a lot… I thought that the uncertainty in those situations was a threat to me and a threat to my profile as a footballer…*

(Participant 2)

*I saw it as a threat… like the uncertainty could expose me. I felt like it was a threat to who I was as a baseball player*

(Participant 8)

On the other hand, after experiencing detrimental uncertainty in their careers, some of the participants managed to look at future uncertain situations as challenges because this uncertainty allowed them to perform better and thrive, as enjoying playing the game was one way that helped them to perceive it differently.

*I found the uncertainty a challenge. I liked it, not knowing, so I had to try my best, and that was fine… If you’re already anxious when you approach uncertain situations, you can find them very threatening, whereas if you’re coming into the game without anxiety, then you can feel excitement and nerves for a game, but it’s not threatening.*

(Participant 5)

#### 4.1.4. Uncertainty as a Lack of Information

Some of the participants chose to focus on the element of available information when they described the situations that were uncertain to them. Not having enough information about an opponent can make athletes feel uncertain about their competition because they do not know what to expect from them or how to respond. Another example derived from the lack of information they experienced when they were sent onto the pitch and did not understand their role in the team or what was expected of them. This lack of information, whether about their role in the team or how to prepare for a competition, might cause them to experience the situation with uncertainty.

*If you’re uncertain about something, you don’t know what the outcome of a process or a situation will be due to not having sufficient information.*

(Participant 1)

*They never give an instruction… This is what we need… We don’t want you to just kick… It probably would have helped had I got direction at the beginning of what they wanted from me.*

(Participant 2)

#### 4.1.5. Uncertainty in Decision-Making

According to the participants, sport involves making multiple decisions in training, during competitions, and even away from the competitive arena. Some participants perceived uncertainty, particularly when making decisions, as it causes them to hesitate and even pass on the responsibility to another teammate. The participants described scenarios during a game where they had to choose one action among several possibilities that could affect the team and the outcome of the game. Feeling uncertain impaired their decision-making since the situation involved possible consequences with an uncertain effect on the game. In uncertain moments, the conscious process of decision-making could cause them to pick the wrong option.

*There can be a lot of uncertainty regarding decision-making within specific actions within the game… I was trying to weigh it up in my head in a split second, the benefits and consequences of my action… I would hesitate and look for a teammate, and I was handing the responsibilities away.*

(Participant 2)

*There’s so much uncertainty in the game… you’re constantly making quick decisions, and I’d overthink it…what’s the right move, what could go wrong.*

(Participant 7)

The participants perceived uncertainty in decision-making during competitive situations, and a lack of information was an integral part of that perception. Since uncertainty constitutes a significant obstacle to effective decision-making, some studies have tried to conceptualize uncertainty from the perspective of decision-makers, wherein a lack of sufficient information is frequently related to uncertainty (Lipshitz & Strauss, 1997; Magnaguagno et al., 2022). In the context of action, uncertainty impacts the decision-maker in the sense of doubt that causes individual differences in reactions (van den Heuvel et al., 2014). In sport contexts, recent work further suggests that athletes continuously integrate incomplete and evolving information under time pressure, shaping both perception and action selection (Gredin et al., 2020; Beck et al., 2025). In terms of sport, actions are a significant part of the game, and the participants differed in their perceptions of the term and its effects on their decision-making. The participants elaborated on their ways of searching for information and talked about weighing up pros and cons or hesitating when required to make a decision in a game due to uncertainty and the unknownness of the outcome. These descriptions are similar to the five heuristic coping strategies utilized by decision-makers in order to cope with uncertainty proposed by Lipshitz and Strauss (1997): reduction through information search; assumption-based reasoning to fill in missing information; weighing pros and cons in order to derive subjective expected utility of options; forestalling to prepare for worst-case scenarios; and suppressing uncertainty in order to ignore doubts and conflicting information.

The participants’ descriptions of using some of these heuristics are in line with the literature that suggests they are used “as a cognitive method for reducing complex decision-making problems in ambiguous, time-pressured or uncertain situations, to reduce the systematic processing of information” (van den Heuvel et al., 2014, p. 26). Recent sport-specific research further supports the use of adaptive heuristics under uncertainty, highlighting how athletes rely on simplified decision rules when processing demands exceed cognitive capacity (Magnaguagno et al., 2022). There are many similarities between military and sports tasks as individuals are required to perform in a complex and dynamic environment, even when only partial or incomplete information is available (Ward et al., 2008). Police officers tend to respond similarly in situations characterized by high risk and uncertainty, as they seek additional information to reduce the level of uncertainty (van den Heuvel et al., 2014). An example drawn from sports psychology literature comes from Abraham and Collins’ (2015) study on coaches’ judgement and decision-making. According to the researchers, when facing an uncertain situation where a decision needed to be made, coaches attempted to gain additional information to understand the problem further. The purpose of this strategy is to reduce uncertainty by searching for more information to use as a resource when making a decision (Lipshitz & Strauss, 1997). The authors suggested that in order to promote more professional decision-making among coaches, they should deliberately be placed in positions of uncertainty during their education, as this will be crucial for their development (Abraham & Collins, 2015).

### 4.2. Mediators of Uncertainty

The perceptions of uncertainty vary between the participants, as do the mediators they suggested to make them feel uncertain. The interviews displayed several themes that could generate uncertainty in the eyes of the athletes. This part describes three themes as potential reasons for perceiving a situation as uncertain: experience, reasons for playing the game, and lack of control.

#### 4.2.1. Experience

The participants mentioned their experience as an overarching mediator of uncertainty. This theme includes the participants’ young age and the fact that they are dealing with uncertain situations for the first time, the familiarity of the task (e.g., competition, trials), which is itself related to their expectations of their own performance, their opponents’ ability, and the tasks’ demands. A lack of information, mentioned above as perceived uncertainty, differs from experience in that athletes perceive the situation as uncertain due to insufficient information in the moment.

The athletes felt uncertain when faced with ambiguous moments in competitions and trials and after injuries, since they were experiencing uncertainty in a sporting context for the first time. As athletes gain experience, they learn and reflect on their responses in similar uncertain moments. Once they have already faced uncertain situations and responded, they can use such responses when experiencing uncertainty in the present. Experience, in their view, is an essential part of dealing with struggles and difficulties, as individuals can develop coping skills to deal with uncertain situations over time based on similar situations and their past behaviour. This is the case, for example, with athletes facing an injury for the first time and not knowing when they can resume playing.

*I have never done anything internationally… That was actually the first international competition I went to…*

(Participant 1)

*I’ve never broken a bone; I’ve been so lucky. I suppose I didn’t really have coping skills in place… especially when you first experience something, it’s sort of traumatic…*

(Participant 3)

Like inexperience, the participants considered a situation’s unfamiliarity and their incapability to set expectations regarding this situation as uncertainties. Since they have not played against many opponents and participated in few competitions or trials, not having enough experience might have affected their expectations regarding those moments. When uncertainty arose, they did not know how they were going to be evaluated or how to respond. They described two dimensions of unfamiliarity: their familiarity with their opponent (i.e., their abilities) and the demands of the competition (e.g., the type of tasks they needed to complete to succeed). These disrupted their preparation for competitive situations and perhaps caused ambiguity and a sense of uncertainty.

*I had no idea what to expect—I only played after school, and it was a very professional environment.*

(Participant 5)

*There’s quite a big gap compared to what they expect from you at the top level…*

(Participant 3)

#### 4.2.2. Reasons for Playing

As part of the interview, the participants discussed essential factors that caused them to keep playing and helped them deal with uncertainty. First, some of the participants talked about factors that increased their level of anxiety in uncertain situations, such as their perceptions of others regarding their abilities and success in competition. When thinking about being judged by managers, peers and even friends or family, athletes’ level of uncertainty tends to increase. Social evaluation and social comparison are vital components of uncertainty from some of the participants’ perspectives and are crucial parts of feeling uncertain directly or indirectly through the emotions accompanying uncertainty.

*I cared about what other people thought of me to an extent… You want a reputation, you want to be popular, I worried I’d lose popularity… I was feeling judged… The social side of the game and the weight of expectation and letting people down was probably the biggest struggle for me.*

(Participant 3)

On the other hand, when facing uncertainty, positive values could protect the athlete from emotions and thoughts linked to uncertainty (e.g., anxiety, pressure). For example, being a part of a team, enjoying competing in the sport they have been playing since childhood, and demonstrating their abilities could decrease their level of uncertainty and negative ruminations regarding its implications.

*Keep remembering why you’re doing it … because you enjoy playing, and you want to be here… Remembering why you’re doing it and that it’s supposed to be fun takes off some of the pressure.*

(Participant 5)

*There was a lot of uncertainty about whether we could play against those guys… When we were training and preparing for that match, a lot of what was talked about was working for the team, doing all the things we grew up doing.*

(Participant 6)

#### 4.2.3. Lack of Control

Another critical factor that helps create uncertainty, according to the participants, is the lack of control an athlete experiences during a competitive situation. Since the situation is not solely under their control and depends on environmental factors such as managers and referees, their feelings of uncertainty increase. Another example of a lack of control concerned securing future goals, such as signing a contract with a professional team, which could be disrupted due to an injury. Athletes tend to think about contracts as financial security when trying to predict the future, and injuries, which are not wholly under their control, affect the certainty of their careers.

*The uncertainty off the pitch is more external… I can’t control the environment, so that’s where the uncertainty came from.*

(Participant 5)

*I suppose as you get a little bit older and you’ve got money away in the bank… Even then, there’s uncertainty because what if they suffer a career-ending injury at 26 and get nothing else?*

(Participant 4)

Experience and familiarity with a task were found to be mediators of uncertainty. According to the participants, their first experiences of competitions and the fact that they did not know what to expect generated a sense of uncertainty. In the literature, the definition of experience was once thought to be related to the number of years spent participating in a particular sport (Mellalieu et al., 2004). However, further investigation suggested that experience “is the current product of a process whereby knowledge is acquired and adapted so that action, reflection, and learning take place” (Hanton et al., 2007, p. 32). The learning process occurs by being exposed to a variety of incidents and situations, as the athlete needs to adapt their perceptions of and preparations for future events (Hanton et al., 2007). More recent work reinforces this dynamic view of experience, suggesting that athletes’ psychological adaptation to competitive environments is shaped not only by exposure but also by how they interpret and make sense of these experiences over time (e.g., Dithurbide et al., 2025). According to this perspective, being exposed to uncertain moments in sport may form a critical part of the learning process, as athletes gradually recalibrate their expectations, interpretations, and responses to ambiguity.

This distinction is important for differentiating between experience and lack of information. While the latter reflects situational uncertainty, the former relates to an athlete’s capacity to interpret and respond to uncertainty based on prior exposure. In line with this, recent findings indicate that more experienced athletes tend to appraise uncertain situations differently, often perceiving them as more manageable or even as opportunities for growth, rather than purely as sources of threat (e.g., Poucher et al., 2023). Thus, a shortage of prior exposure to uncertainty may increase the likelihood that athletes experience uncertainty as disruptive or overwhelming.

Moreover, the experience level of a sportsperson is related to interpretations of anxiety as part of the process of being exposed to adverse environments (Mellalieu et al., 2004). After experiencing an uncertain situation, athletes may become more familiar with competitive environments and cope more adaptively in subsequent situations, as reflected in the participants’ accounts. This process is achieved through reflection on past events and the reappraisal of thoughts and emotions (Hanton et al., 2007), but also through broader processes of psychological adjustment within the sport environment, including perceived support, expectations, and cultural norms (Poucher et al., 2023). These findings further highlight that coping with uncertainty is not only an individual cognitive process but is embedded within the broader sport context.

Another theme that was mentioned by the participants regarding perceptions of uncertainty was perceived control. The athletes described this as the inability to control external factors, which increased their sense of uncertainty. On the other hand, when coping with uncertainty, focusing on controllable aspects was a central strategy for reducing both uncertainty and accompanying anxiety. Regarding perceived control, it is argued by Jones and Hanton (1996) that perceptions of control over the self and the environment are critical for both anxiety regulation and performance. Anxiety is closely related to uncertainty in sport (Martens et al., 1990), and it has been suggested that athletes’ perceived lack of control in uncertain situations increases their anxiety (Hanton & Connaughton, 2002). Recent research further supports this link, showing that athletes’ mental health and emotional responses are strongly influenced by their perceived ability to manage and control demands within the sport environment (Poucher et al., 2023). Importantly, this aligns with the current findings, as participants described shifting their focus toward controllable elements as a way of reinterpreting uncertainty and regulating their responses. Thus, perceived control appears to function not only as a situational factor but also as a psychological mechanism through which uncertainty is appraised, either as threatening or as manageable.

### 4.3. Thoughts and Emotions in Conditions of Uncertainty

#### 4.3.1. Anxiety

The most prevalent and immediate emotion relating to uncertainty in sport is anxiety, according to all of the participants, and several arguments were mentioned as a possible explanation for the generation of anxiety. First, the importance of the competitive situation and even playing the sport they enjoy might cause these athletes to consider the possibility of failure. Anxiety is the final result that the participants experienced, but it was rooted in the uncertainty of the competitive situation that, in turn, made the participants think about the consequences of losing.

#### 4.3.2. Threat to Identity and Self-Doubt

Since sportspeople cannot predict the outcome of an upcoming competition, losing in a game can threaten their self-esteem, which is another way they perceive uncertainty. The harm to their sporting identity was also a reason for experiencing anxiety since this threat was caused by a failure to perform under conditions of uncertainty and may lead them to question their reason for playing and competing. The uncertainty in situations such as a transfer to a better team and the accompanying pressure to prove their abilities, expectations from others, and social judgement made the participants feel a lack of confidence. Uncertainty in trials made the participants feel nervous because if they did not handle them well, their failure made them feel self-doubt and implied that they were not good enough.

*Anxiety is the first thing that comes up… then there’s the fear of losing, hurting your ego, and what people will think about you.*

(Participant 7)

*The uncertainty made me question things… Do I want to play the sport? Who am I? … If I didn’t play the sport, what does that mean for me? And the anxiety that accompanies those sorts of questions, the experience of nerves, all that together internally … probably didn’t help.*

(Participant 3)

Importantly, responses to uncertainty are not shaped solely by cognitive and emotional processes but also by perceived social costs. Classic work by Bar-Eli et al. (2007) demonstrated that soccer goalkeepers tend to dive during penalty kicks rather than remain in the center, despite evidence suggesting that staying central may be a more optimal strategy. This tendency is driven by the social cost of appearing passive or inactive. Such findings suggest that athletes’ responses to uncertainty may also be influenced by concerns related to evaluation, identity, and social perception. This aligns with the current findings, where participants described uncertainty as threatening their self-image and perceived competence.

### 4.4. Coping with Uncertainty

This last part of the results section outlines athletes’ coping skills and the ways they deal with uncertainty in order to continue playing and maintain excellent levels of performance. The participants mentioned several factors that helped them cope with uncertainty: communication, gathering information, social support, focusing on the controllable, and growth out of dealing with uncertainty.

#### 4.4.1. Communication

All the participants mentioned communication as a coping skill, whether they played as individuals or in group sports, when faced with uncertainty during a competitive event: delivering messages between teammates, coaches and other staff that mostly involved instructions or relaxing content regarding the uncertain situation. One of the examples mentioned was an uncertain competitive situation in which an opponent played in a way for which they were unprepared. The participants found that a way of dealing with this uncertainty was to communicate with deliberate instructions, whether with their teammates or the coach, to increase confidence by sharing thoughts, feelings, and information with others. According to the participants, in group sports, communication is a significant coping skill when dealing with uncertainty and the accompanying anxiety.

*You might talk to your teammates and mention they’re now playing an extra player in the middle … so we need to change to this tactic… You try to get the behaviours on your team to change to kind of deal with the uncertainty.*

(Participant 5)

*Communicating with the players immediately around you. Kind of talking throughout the team… The coach probably gets communication too… Just talking to the people around you is good.*

(Participant 6)

#### 4.4.2. Gathering Information

As mentioned earlier, uncertainty is perceived as insufficient information regarding a current or future event. In order to deal with that, the participants talked about a process of information gathering, whether direct or indirect. Knowing more information about their next opponent, their strengths and weaknesses, or even what their tactical plan is can contribute to decreasing uncertainty. One athlete described a structured method, suggested by his coach, that included note-taking on the day of a competition. The athlete went through the circuit after his fights and collected data about future rivals, adding it to a bank of information that included strengths, weaknesses, and key factors about his opponents that needed to be addressed before his next competition.

*You watch everyone else so you can get an idea of how good they are, what tactics they use … potential weaknesses you can use to come up with a plan… It’s a process of gathering information… through that, uncertainty decreases.*

(Participant 1)

*Knowing more information about your opponent makes the game less uncertain.*

(Participant 5)

#### 4.4.3. Social Support

Some participants mentioned social support as a critical factor for dealing with uncertainty. Whether formed by parents, friends or teammates, social support helped the athletes to share their thoughts and feelings when difficulties, such as uncertainty, occurred. One of the participants described their support system as crucial when trying to deal with an injury. Their uncertainty about that injury was not knowing how severe it was and how long their recuperation would be. When another participant transferred to a better team, he faced the uncertainty of not knowing how well he would fit into the team and whether he was going to play. However, long drives with his father spent sharing his concerns and expressing them allowed him to relax.

*From the moment we left my door and got in the car, we talked about football the whole way, whether about my team or just anything, and I think that sort of relaxed me because I always had my dad to speak to. I always spoke to him.*

(Participant 4)

*The uncertainty of the duration of my recovery. It was an uncomfortable experience… I had the most phenomenal support system… My family’s incredible… I know my support system helped me through the times of uncertainty more than anything else.*

(Participant 3)

#### 4.4.4. Focusing on the Controllable

A lack of control is one of the mediators, according to the participants, that can increase the likelihood of perceived uncertainty. The participants argued that trying to focus on things that are in their control can be used as a skill for coping with uncertainty. Focusing on their own abilities instead of thinking about how good their opponent is was used and found to be beneficial, and the athletes described it as part of their athletic identity. Other examples given included trying to ignore the external pressure and worry by acknowledging their strengths in preparation for a competition or remembering they enjoy playing. When focusing on things that are out of their reach, uncertainty and worry grow and can affect sporting performance. A positive solution would be to focus on the present and the factors that are under the athletes’ full control.

*When you go into those maybe uncertain or unfamiliar environments, you can focus on “your bread and butter”, what you’re good at.*

(Participant 6)

*I can deal with the things I’m uncertain about within me so I can try and control all of these things that are within my ability to control… There’s no point worrying about things you can’t control.*

(Participant 1)

#### 4.4.5. Growth out of Dealing with Uncertainty

After discussing uncertainty in sport and the ways they coped with it, the participants were asked about the opportunity to eliminate it from sport if, hypothetically, they could. Except for one participant, every athlete talked about uncertainty as an integrated part of their sport, the benefits of it (e.g., uncertainty enables every athlete to have a chance and win), and the fact that the process of dealing with uncertainty made them stronger. When facing uncertainty, the athletes experienced difficulties and self-doubt and questioned whether they were competent enough to become professional athletes. After they managed to overcome the consequences of uncertain moments in their career, the participants felt more competent and mentally stronger.

*Uncertainty is what made me; it’s what kept me in the team even though it was my threat… It forced me to go back and train hard. That was all because of uncertainty.*

(Participant 2)

*Uncertainty is such an important characteristic of the game, of sport itself… I’m not sure anyone would engage in sport if it didn’t have a little bit of uncertainty or a bit of anxiety about it… It says something about you if you can overcome it—strength of character, physical strength.*

(Participant 3)

The participants mentioned communication as a significant factor in dealing with uncertainty, as their coach and teammates are part of this coping skill. The importance of communication to team cohesion, coordination between players and level of performance is known in sports literature (e.g., Eccles & Tenenbaum, 2004; Lausic et al., 2009). General psychology studies have also acknowledged the contribution of group communication to reducing uncertainty through behaviours that seek information and express emotions regarding uncertainty (Kramer, 2014). Berger (1979) recognized three different communication strategies between co-workers for reducing uncertainty. Whether passively or actively, individuals may interact with a trusted peer and make some comparisons based on observations or even directly ask for information to reduce their level of uncertainty. Similarly, the participants mentioned social comparison and observation as a pre-phase to communicating with teammates and staff. Once uncertainty has been experienced, individuals attempt to manage their uncertainty using a process of communication with other members of the group (Kramer, 2014). Once again, the relationship with perceived uncertainty as a lack of information is related to the coping skill used.

Other than the use of information, individuals can use internal processes to reduce uncertainty without interacting with others (Kramer, 2014); for example, cognitive processes such as focusing on controllable elements rather than the environment and trusting one’s own abilities. These internal cognitive processes attempt to reduce uncertainty in that they are not trying to prevent the experience of uncertainty but to manage it once it has already been experienced (Kramer, 2014). The distinction identified in the present study between conscious deliberation and more intuitive responses aligns with research suggesting that conscious decision-making may, in some cases, lead to less optimal outcomes compared to more automatic processes (Trommershäuser, 2009). Under conditions of uncertainty, excessive cognitive control may interfere with well-learned sensorimotor processes. In this context, the coping strategy described by participants as “trusting one’s abilities” may reflect a shift toward more automatic, experience-based responding, which can facilitate performance in dynamic and time-constrained environments.

The use of social support and significant others has a lot in common with communication, but it is used after the uncertainty has occurred. Further, the role of the significant other in the psychological development of elite athletes is consistent with the talent development literature (e.g., Gould et al., 2002; Woodhead et al., 2024). Considering the relatively young age of the participants in the current study, social support is even more important. Social support could assist in coping with uncertainty in several ways. First, it facilitates the experience of uncertainty through messages and behaviours that could increase certainty; those offering support provide a stable relationship through validation, instrumental support, and acts that project the availability of support (Brashers, 2001). In addition to dealing with uncertainty, significant others, in the form of parents, coaches, and older, more experienced competitors, could facilitate dealing with anxiety symptoms by explaining how they dealt with it or interpreted it in a positive way (Hanton et al., 2007). Supportive others can also influence uncertainty management processes by providing direct or indirect assistance as sources of information (Brashers, 2001).

## 5. Conclusions

The present study aimed to explore how athletes perceive and cope with uncertainty in sport, addressing a level of analysis that has received relatively limited attention, namely the subjective and lived experience of uncertainty. The thematic analysis identified five higher-order themes: (a) inability to predict future events, (b) internal versus external sources of uncertainty, (c) perception of uncertainty as a threat or challenge, (d) uncertainty in decision-making, and (e) lack of information as a source of uncertainty. These themes provide insight into how uncertainty is experienced by athletes beyond observable performance outcomes.

The first theme, uncertainty as an inability to predict future events, is consistent with foundational definitions of uncertainty in decision-making literature, which emphasize unpredictability and probabilistic reasoning (Kahneman & Tversky, 1973; Downey & Slocum, 1975). However, the present findings extend these conceptualizations by demonstrating how unpredictability is experienced subjectively by athletes, often accompanied by emotional and identity-related concerns.

The distinction between internal and external sources of uncertainty aligns with prior work in motor decision-making and action control, which differentiates between internally generated ambiguity and externally imposed uncertainty (Krüger & Hermsdörfer, 2019). The current findings build on this distinction by showing that athletes not only experience these sources differently but also interpret and respond to them in ways that influence their performance and psychological state. The perception of uncertainty as either a threat or a challenge reflects well-established appraisal processes in sport psychology (Lazarus & Folkman, 1984; Martens et al., 1990), whereby individuals interpret competitive situations in ways that shape emotional responses such as anxiety. Recent sport-specific research further supports this distinction, demonstrating that competitive stressors may be appraised as either debilitative or facilitative depending on athletes’ interpretations (e.g., Uphill et al., 2019). Within this framework, uncertainty itself does not inherently impair performance; rather, its impact depends on whether it is perceived as overwhelming or as an opportunity for engagement and growth. The present study extends this literature by illustrating how these appraisals emerge specifically in the context of uncertainty, highlighting the role of subjective interpretation in shaping athletes’ responses.

The themes of uncertainty in decision-making and lack of information further support existing literature suggesting that uncertainty is closely linked to information processing and action selection (Lipshitz & Strauss, 1997; van den Heuvel et al., 2014). In line with more recent sport science research, athletes appear to rely on contextual information and prior knowledge to reduce uncertainty (Gredin et al., 2020; Magnaguagno et al., 2022). However, the present findings demonstrate that when such information is unavailable or insufficient, uncertainty becomes not only a cognitive challenge but also an emotional and experiential one.

Across themes, uncertainty was experienced as a multifaceted phenomenon shaped by perceived control, prior experience, and situational context. This finding aligns with contemporary perspectives in sport science, suggesting that uncertainty is not solely an objective property of the task but is actively constructed through athletes’ interactions with their environment (Gredin et al., 2020; Beck et al., 2025). In particular, recent work on decision-making in sport highlights that the interpretation and use of information under uncertainty are strongly influenced by prior knowledge and expertise (Magnaguagno et al., 2022), supporting the current finding that athletes’ previous experiences play a central role in shaping how uncertainty is perceived. Furthermore, emerging research has emphasized the role of uncertainty in shaping emotional and predictive processes, including anxiety and anticipatory responses (Harris et al., 2025), which is consistent with the present findings linking uncertainty appraisal to emotional reactions.

From a qualitative perspective, these findings support approaches that emphasize lived experience and meaning-making in sport (Smith & Sparkes, 2016; Vaughan et al., 2022), highlighting that uncertainty is not merely encountered but interpreted, negotiated, and given meaning by athletes.

The coping strategies identified: communication, information gathering, social support, and focusing on controllable aspects, are broadly consistent with existing research on coping and decision-making under uncertainty (Lipshitz & Strauss, 1997; Kramer, 2014). Importantly, recent systematic reviews in sport psychology emphasize that coping in sport is not solely an individual process but is inherently interpersonal and context-dependent, involving dynamic interactions with coaches, teammates, and the broader social environment (Woodhead et al., 2024). This perspective extends the current findings by suggesting that the coping strategies reported by athletes are not merely individual techniques but are embedded within social and relational contexts and shaped by how uncertainty is perceived and negotiated within these environments. Accordingly, the present study highlights how athletes’ interpretations of uncertainty influence not only which coping strategies are used, but also how these strategies are enacted in practice.

Importantly, the findings have implications for the construct of intolerance of uncertainty (IU). IU has been conceptualized as a dispositional tendency to respond negatively to uncertain situations (Carleton, 2012; Koerner & Dugas, 2008), and has been associated with cognitive, emotional, and behavioural responses such as worry, avoidance, and hesitation (Robinson & Freeston, 2015; Thibodeau et al., 2013). The themes identified in the present study—particularly those related to threat appraisal, anxiety, and decision-making hesitation—closely mirror these processes.

However, the findings also suggest that uncertainty in sport is not uniformly negative. Some athletes described uncertainty as motivating or growth-enhancing, indicating that responses to uncertainty may depend on context and individual interpretation. This nuance suggests that existing conceptualizations of IU may require adaptation when applied to sporting environments, where uncertainty may also play a facilitative role. Therefore, the present study serves as an exploratory step toward integrating IU into sport psychology. While the findings suggest conceptual overlap between athletes’ experiences and IU-related processes, further research is required to determine how IU can be operationalized and measured in sport contexts, and whether existing instruments (e.g., IUS-12; Carleton et al., 2007) require adaptation.

Several limitations should be acknowledged. First, the relatively small and homogeneous sample, consisting primarily of former youth athletes, may limit the transferability of the findings. However, within qualitative research, smaller samples are often justified when the aim is to obtain in-depth insights into participants’ experiences, consistent with the concept of information power (Malterud et al., 2016). Second, the retrospective nature of the data may have introduced recall bias, as participants reflected on past experiences rather than reporting in real time. This is a known limitation in qualitative research on lived experiences (Smith & Sparkes, 2016), and future studies may benefit from incorporating real-time or longitudinal designs. Third, while thematic analysis allows for rich interpretation of subjective experiences, it is inherently influenced by researchers’ interpretations and analytic decisions (Braun et al., 2016). Although steps were taken to ensure rigor, including systematic coding procedures, the findings should be understood as one possible interpretation of the data. Finally, although the findings suggest conceptual overlap with IU, the study did not directly measure IU. Future research should examine whether existing IU measures (e.g., IUS-12) are appropriate for sport contexts or require adaptation, and whether IU operates differently in performance environments compared to clinical settings.

Future research may benefit from combining qualitative and quantitative approaches to capture both subjective experiences and behavioural outcomes, despite the known challenges associated with integrating mixed methods designs (Moran et al., 2011). Overall, the findings highlight the importance of understanding uncertainty as both a performance-related and experiential phenomenon, suggesting that integrating these perspectives may provide a more comprehensive understanding of athlete functioning under uncertainty.

Overall, the findings highlight the importance of understanding how athletes experience uncertainty and suggest that developing the capacity to engage with uncertainty may be central to both performance and psychological development in sport.

## Data Availability

The original contributions presented in this study are included in the article/Supplementary Material. Further inquiries can be directed to the corresponding authors.

## Supplementary Materials

The following supporting information can be downloaded at: https://www.mdpi.com/article/10.3390/bs16040616/s1, File S1: List of key questions.
