# Supplementary material for: Understanding How Athletes Manage Uncertainty in Sport
Source: Behav Sci (Basel). 2026 Apr 21;16(4):616. doi: 10.3390/bs16040616 (PMC13112974; doi:10.3390/bs16040616)
Supplement: Supplementary file 1 [file behavsci-16-00616-s001.zip › behavsci-4174573-supplementary.pdf]

List of key questions

1. I want you to tell me when did you start playing…? For example why did you choose/what you loved about that sport.
2. What was your first competition? Can you describe me that day
3. What was your first big accomplishment? Can you describe me that day
4. Can you describe me your planning for a competition? A day before until the fight/game itself
5. There is a theory about dealing with uncertainty in a daily decision and business… do you think that your sport has ambiguous/uncertain situations? Give me an example if you can.
6. When you encounter this of situation, do you think about it during a competition?
7. What feelings do you experience? Give me an example for what going through your mind in that situations.
8. Do you try to prepare yourself to this kind of situations? For example, a routine you do with your coach or discussing about this moments before a competition
9. When you encounter this of situation, what do you do in order to be at your best performance?
10. How can you think athletes in your sport/yourself can better prepare for ambiguous/uncertain situations?
